# Incidence of propofol-related infusion syndrome in critically ill adults: a prospective, multicenter study

**DOI:** 10.1186/cc8145

**Published:** 2009-10-29

**Authors:** Russel J Roberts, Jeffrey F Barletta, Jeffrey J Fong, Greg Schumaker, Philip J Kuper, Stella Papadopoulos, Dinesh Yogaratnam, Elise Kendall, Renee Xamplas, Anthony T Gerlach, Paul M Szumita, Kevin E Anger, Paul A Arpino, Stacey A Voils, Philip Grgurich, Robin Ruthazer, John W Devlin

**Affiliations:** 1 Department of Pharmacy, Tufts Medical Center, 800 Washington Street, mailstop #420, Boston, MA 02111, USA; 2 Department of Pharmacy, Spectrum Health, 100 Michigan Street NE (MC01), Grand Rapids, MI 49503, USA; 3 Department of Pharmacy Practice, Massachusetts College of Pharmacy and Health Sciences, 19 Foster Street, Worcester, MA 01608, USA; 4 Division of Pulmonary, Critical Care and Sleep Medicine, Tufts Medical Center, 800 Washington Street, Boston, MA 02111, USA; 5 Department of Pharmacy, Mayo Clinic College of Medicine, Mayo School of Health Sciences, Siebens Medical Education Building 11200 First Street SW, Rochester, MN 55905, USA; 6 Department of Pharmacy, Boston Medical Center, 1 Boston Medical Center Place, Boston, MA 02118, USA; 7 Department of Pharmacy, University of Massachusetts Memorial Medical Center, 119, Belmont Street, Worcester, MA 01605, USA; 8 Department of Pharmacy, Concord Regional Hospital, 250 Pleasant Street, Concord, NH 03301, USA; 9 Department of Pharmacy Practice, John H. Stroger Jr. Hospital of Cook County, 1901 W. Harrison Street, Chicago, IL 60612, USA; 10 Department of Pharmacy and Center for Critical Care, The Ohio State University Medical Center, 410 West 10thAvenue, Columbus, OH 43210, USA; 11 Department of Pharmacy Services, Brigham and Women's Hospital, Pharmacy Administration L-2, 75 Francis Street, Boston, MA 02115, USA; 12 Department of Pharmacy, Massachusetts General Hospital, 55 Fruit Street, Boston, MA 02114, USA; 13 Stacey Voils, Department of Pharmacy, Virginia Commonwealth University Health System, 410 North 12thStreet, Richmond, VA 23298, USA; 14 Institute for Clinical Research and Health Policy Studies, Biostatics Research Center, Tufts Medical Center, 35 Kneeland Street, Boston, MA 02111, USA; 15 Northeastern University School of Pharmacy, 360 Huntington Avenue, Mugar 206, Boston, MA 02115, USA

## Abstract

**Introduction:**

While propofol is associated with an infusion syndrome (PRIS) that may cause death, the incidence of PRIS is unknown. Determining the incidence of PRIS and the frequency of PRIS-related clinical manifestations are key steps prior to the completion of any controlled studies investigating PRIS. This prospective, multicenter study sought to determine the incidence of PRIS and PRIS-related clinical manifestations in a large cohort of critically ill adults prescribed propofol.

**Methods:**

Critically ill adults from 11 academic medical centers administered an infusion of propofol for [>/=] 24 hours were monitored at baseline and then on a daily basis until propofol was discontinued for the presence of 11 different PRIS-associated clinical manifestations and risk factors derived from 83 published case reports of PRIS.

**Results:**

Among 1017 patients [medical (35%), neurosurgical (25%)], PRIS (defined as metabolic acidosis plus cardiac dysfunction and [>/=] 1 of: rhabdomyolysis, hypertriglyceridemia or renal failure occurring after the start of propofol therapy) developed in 11 (1.1%) patients an average of 3 (1-6) [median (range)] days after the start of propofol. While most (91%) of the patients who developed PRIS were receiving a vasopressor (80% initiated after the start of propofol therapy), few received a propofol dose >83 mcg/kg/min (18%) or died (18%). Compared to the 1006 patients who did not develop PRIS, the APACHE II score (25 +/- 6 vs 20 +/- 7, *P *= 0.01) was greater in patients with PRIS but both the duration of propofol use (*P *= 0.43) and ICU length of stay (*P *= 0.82) were similar.

**Conclusions:**

Despite using a conservative definition for PRIS, and only considering new-onset PRIS clinical manifestations, the incidence of PRIS slightly exceeds 1%. Future controlled studies focusing on evaluating whether propofol manifests the derangements of critical illness more frequently than other sedatives will need to be large. These studies should also investigate the mechanism(s) and risk factors for PRIS.

## Introduction

Propofol has been commonly used as a sedative in the intensive care unit (ICU) for more than 20 years and, when prescribed based on product labeling recommendations, is generally considered safe [1]. Nevertheless, a troubling syndrome known as the propofol-related infusion syndrome (PRIS) exists, which was first reported 17 years ago in five pediatric ICU patients who developed metabolic acidosis, bradyarrhythmias and progressive myocardial failure after receiving propofol at a high dose [2]. Since this initial report, 78 additional cases of PRIS have been published with a mortality rate exceeding 80% [3-57]. In addition, a recent analysis of the Food and Drug Administration (FDA) MEDWATCH system identified a further 1139 suspected cases of PRIS that were associated with 30% mortality [58]. Postulated risk factors for PRIS include use of a high propofol dose (>83 μg/kg/min), a duration of therapy less than 48 hours and concomitant vasopressor therapy [59-61].

Despite an increasing awareness among clinicians regarding this syndrome, and the large number of recent publications surrounding it, a number of unresolved questions exist. For example, the clinical manifestations that make up PRIS remain vague because many of these reflect either common pharmacologic manifestations of propofol (e.g. bradycardia) or common manifestations of critical illness (e.g. metabolic acidosis) [60,61]. Furthermore, the incidence of PRIS among critically ill adults is currently unknown given the voluntary nature by which adverse events are reported to the FDA MEDWATCH system, the propensity for only those cases associated with a poor outcome (e.g. death) to be reported and the total number of patients exposed to propofol that these PRIS cases represents is unknown [58].

Determining the incidence of PRIS and the frequency of PRIS-related clinical manifestations in a large cohort of critically ill patients is a crucial step when designing large, controlled studies investigating PRIS (e.g., propofol vs. non-propofol regimens). We therefore sought to: identify the incidence of PRIS in a large cohort of critically ill adults receiving propofol for more than 24 hours; determine the frequency by which individual PRIS clinical manifestations and risk factors occur; and estimate sample size requirements for future controlled studies surrounding PRIS.

## Materials and methods

This prospective, observational study was approved by the Institutional Review Board at each of the 11 academic medical centers where it was conducted and the need for informed consent was waived at each site. From 1 April to 30 November, 2008, adults admitted to an ICU and treated with propofol for at least 24 hours were evaluated. Patients were excluded if they had rhabdomyolysis (creatinine phosphokinase (CPK) ≥ 10,000 IU/L) prior to propofol exposure, an admission history of familial mitochondrial disease, a prognosis considered to be hopeless by the admitting physician or who had prior exposure to propofol during the current hospital admission.

At the time of enrollment, the following baseline demographic data was collected: age, gender, past medical history, ICU admitting service, primary ICU admitting diagnosis and the Acute Physiology and Chronic Health Evaluation (APACHE) II score at ICU admission [62]. The specific PRIS-associated clinical manifestations and risk factors used in this study were identified from PRIS published case reports [2-57]. These case reports were identified from a MEDLINE search (1980 to December 2007), using the following search terms: propofol, propofol infusion syndrome, propofol-related infusion syndrome, PRIS, rhabdomyolysis and adverse drug events. This strategy is similar to that used in a recent evaluation of the FDA MEDWATCH database [58].

Based on the above analysis, PRIS-associated clinical manifestations were grouped under nine categories and defined as follows: rhabdomyolysis (CPK ≥ 10,000 IU/L); hypotension (systolic blood pressure ≤ 90 mmHg or current use of a vasopressor agent); hepatic transaminitis (increase in the aspartate aminotransferase and/or alanine aminotransferase ≥ 3 times above baseline); metabolic acidosis (arterial pH ≤ 7.30 along with a serum bicarbonate ≤ 18 mg/dL); hypertriglyceridemia (serum triglyceride concentration ≥ 400 mg/dL); hypoxia (partial pressure of arterial oxygen ≤ 60 mmHg); hyperthermia (temperature ≥ 38.3°C); cardiac dysfunction that included a Brugada-like ECG pattern, asystole, pulseless electrical activity, ventricular fibrillation, sustained ventricular tachycardia of 30 seconds or longer, myocardial failure (ejection fraction ≤ 40%), or bradycardia (heart rate ≤ 50 bpm not felt to be related to a medication other than propofol); and renal failure that included oliguria (urine output ≤ 0.5 mL/kg/hr for ≥ 6 hours), anuria (urine output ≤ 10 mL/hr for ≥ 6 hours), elevation in serum creatinine (increase of ≥ 1 mg/dL from baseline), or hyperkalemia (serum K^+ ^≥ 6 mg/dL (excluding other known causes or hemolyzed specimens)). A patient was deemed to have experienced a particular manifestation category if they experienced any manifestation within the category. The presence of known risk factors for PRIS (i.e., a high propofol dose (= 83 μg/kg/min (5 mg/kg/hr)) at any time point and concomitant vasopressor therapy) were also identified [59-61].

Patients were monitored daily for the presence of each PRIS manifestation and risk factor by an experienced critical care pharmacist at baseline, during the period of propofol administration (up to 30 days), and then for 24 hours after propofol was discontinued. The presence of each PRIS clinical manifestation predefined by specific clinical and laboratory values was based on the worst value for each value in the prior 24 hours. Although all progress notes, ICU flow sheets and medication administration records were reviewed, use of additional laboratory testing and/or diagnostic assessment outside of that which occurred as a part of routine clinical practice was not mandated as part of the study. The pharmacists who collected data were instructed not to share any information related to the data they collected with the clinical team nor make interventions pertaining to propofol therapy. All data were recorded on a study case report form and then entered into a secure web-based database.

For the purposes of this study, PRIS was defined as the development of metabolic acidosis and cardiac dysfunction along with at least one of rhabdomyolysis, hypertriglyceridemia or renal failure after the initiation of propofol therapy. This definition was based on a review of 83 published reports of PRIS, incorporated each pertinent PRIS-associated clinical manifestation listed above and was finalized through investigator consensus. The presence of hypotension, hepatic transaminitis, hypoxia and hyperthermia were not included in the PRIS definition given the low incidence by which they are reported in published case series and the fact that they are commonly observed in the critically ill. Other analyses compared demographic factors, the duration of both propofol use and ICU stay, and patient outcome between PRIS and non-PRIS patients. Additionally, the number of new-onset PRIS-related clinical manifestations experienced per patient, the manner and timing by which the PRIS definition was met, the frequency of each PRIS-related clinical manifestation and the number of PRIS-related clinical manifestations for each day of therapy was determined.

Patient characteristics and outcomes were expressed as mean ± standard deviation, median and interquartile range (IQR) or percent where appropriate. Comparisons between groups were performed using the Student's t-test, Mann-Whitney U test or the chi-squared test with the Yates correction where appropriate. A *P *value of less than 0.05 was considered significant for all analyses. All statistical analyses were performed using SPSS 16.0 (SPSS, Chicago, IL, USA).

## Results

Among the 1017 patients followed, 1.1% (11/1017) developed PRIS as it was defined for the purposes of the study (i.e., development of metabolic acidosis and cardiac dysfunction along with at least one of: rhabdomyolysis, hypertriglyceridemia or renal failure after the initiation of propofol therapy). The development of metabolic acidosis, cardiac dysfunction and renal failure after the start of propofol therapy accounted for the definition of PRIS being met in all patients where PRIS was identified. One of the PRIS patients also developed hypertriglyceridemia. None of the patients who met our definition for PRIS had rhabdomyolysis nor did their cardiac dysfunction consist of a Brugada-like ECG pattern. Most (91%) of the 11 PRIS patients received vasopressor therapy. In 80% of cases, vasopressor therapy was initiated after propofol therapy was started. Few of the PRIS patients (18%) were administered a dose of propofol that exceeded 83 μg/kg/min at any point over the course of therapy.

**Figure 1:**
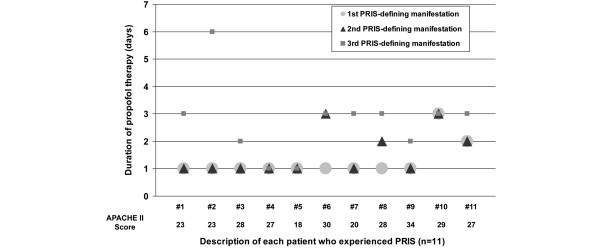
Timing of each PRIS-defining clinical manifestation relative to the start of propofol therapy initiation and admission APACHE II score among the 11 patients who developed PRIS.  APACHE = acute physiology and chronic health evaluation; PRIS = propofol-relation infusion syndrome.

Relative to the start of propofol therapy, the first two PRIS-defining clinical manifestations (i.e. metabolic acidosis, cardiac dysfunction, or renal failure), on average, occurred at [median (range)] 1 (1 to 3) days with the third (and defining) PRIS clinical manifestation occurring at a median of 3 (1 to 6) days (Figure 1). Two of the 11 patients with PRIS experienced all three PRIS-defining clinical manifestations on the first day after propofol was started with 10 of 11 patients experiencing all three manifestations within three days. Among the 11 patients only patients number 6 and number 11 died and only patients number 6 and number 8 were exposed to a propofol dose exceeding 83 μg/kg/min. Demographic variables and clinical outcomes were similar between the 11 patients who experienced PRIS and the 1006 patients who did not with the exception that the 11 patients with PRIS had a higher APACHE II score at ICU admission (*P *= 0.03) and were more likely to be admitted to a surgical service other than trauma or neurosurgery (*P *= 0.04; Table 1)

**Table 1:** Comparison of demographic variables and clinical outcomes between PRIS and non-PRIS patients

	PRIS n = 11	No PRIS n = 1006	*P *value
Age (years)^A^	58 ± 14	56 ± 18	0.64

Male (%)	82	65	0.4

APACHE II score^A^	25 ± 6	20 ± 6	0.03

Admitting service (%)			

Medicine	36	35	0.82

Surgery			

Neurosurgery	9	25	0.39

Trauma	9	23	0.47

Other surgery	46	17	0.04

Primary reason for ICU admit (%)			

Trauma	18	23	1.0

Surgery	28	21	0.89

Neurological	9	20	0.6

Respiratory failure	36	17	0.2

Cardiac	0	9	0.61

Other	9	10	0.77

Past medical history (%)			

Coronary artery disease	27	30	0.89

Malignancy	36	18	0.24

Congestive heart failure	18	18	0.71

Diabetes	18	16	0.83

Chronic obstructive pulmonary disease	27	10	0.12

Other	9	12	0.87

Use of propofol dose >83 μg/kg/min (%)	18	10	0.68

Duration of propofol (days)^B^	5 (3-7)	4 (3-7)	0.43

Number of PRIS clinical manifestations^C^	5 (2-7)	1 (0-6)	0.0001

Duration of ICU stay (days)^B^	14 (10-18)	12 (7-20)	0.4

ICU mortality (%)	18	20	0.82

Hospital mortality (%)	18	21	0.88

APACHE = acute physiology and chronic health evaluation; ICU = intensive care unit; PRIS = propofol-relation infusion syndrome.

^A^Mean ± standard deviation

^B^Median (interquartile range)

^C^Median (Range)

**Figure 2:**
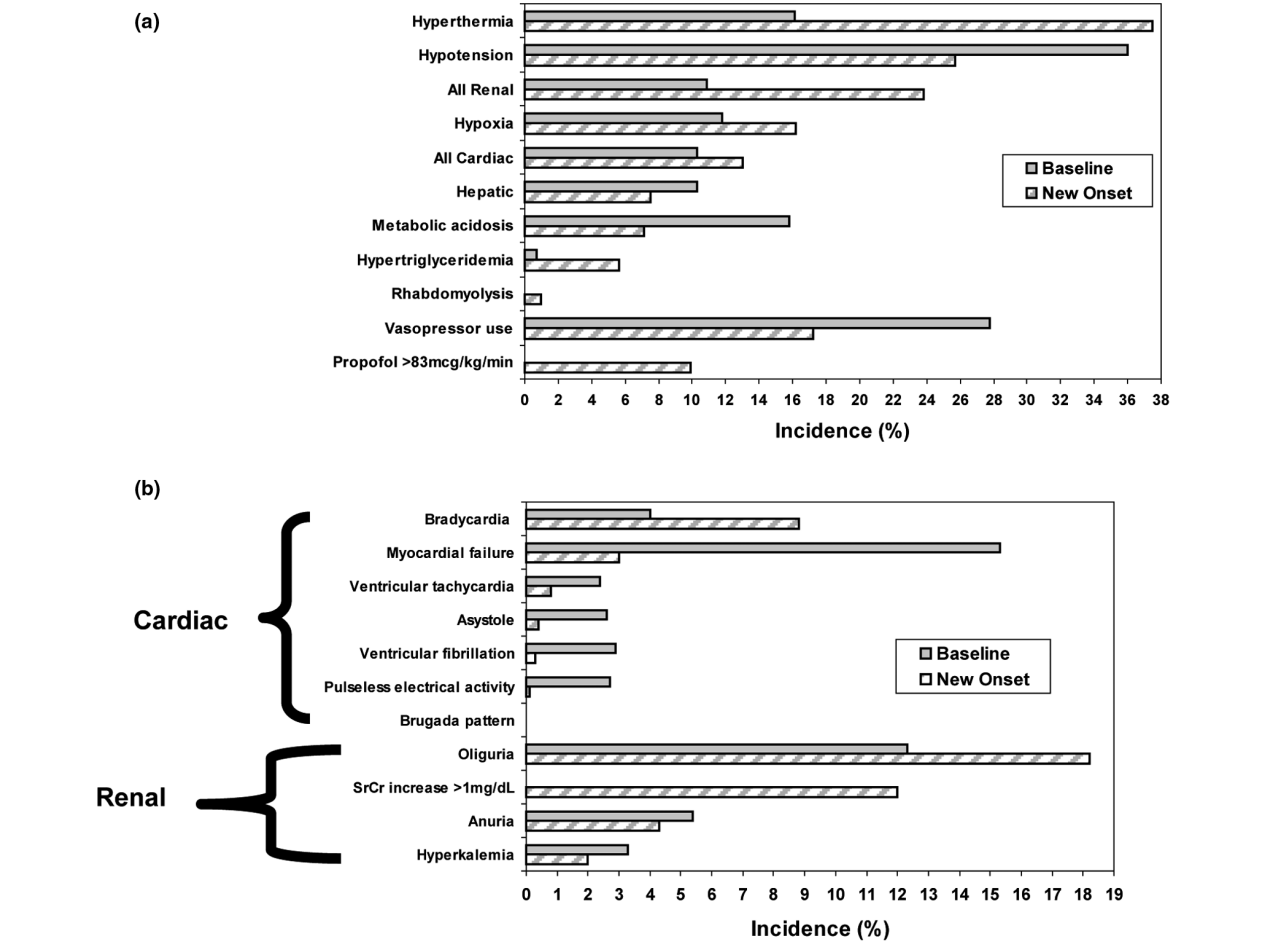
PRIS clinical manifestations.  **(a) **Frequency of PRIS clinical manifestations and risk factors among all patients receiving propofol (n = 1017). **(b) **Frequency of specific cardiac and renal PRIS clinical manifestations among all patients receiving propofol (n = 1017). PRIS = propofol-relation infusion syndrome.

**Figure 3:**
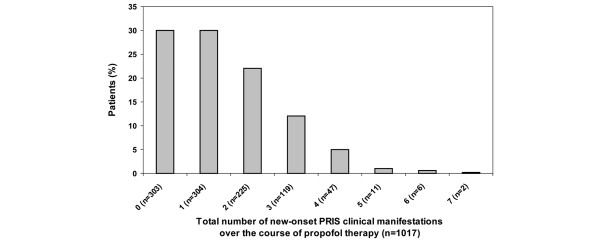
Total number of new-onset PRIS clinical manifestations among all patients receiving propofol (n = 1017).  PRIS = propofol-relation infusion syndrome.

**Figure 4:**
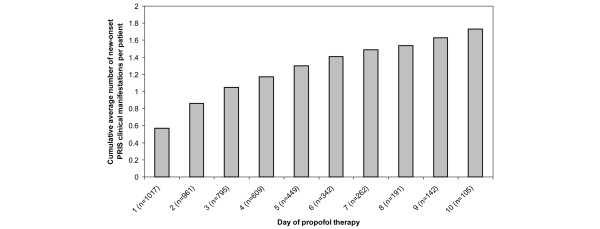
Cumulative average number of new-onset PRIS clinical manifestations per patient by the day of propofol therapy received among all patients receiving propofol (n = 1017).  PRIS = propofol-relation infusion syndrome.

The frequency of each PRIS-associated clinical manifestation, stratified by whether it was present at baseline or developed after the start of propofol therapy, is presented in Figure 2a. In addition, the frequencies of the individual cardiac and renal PRIS clinical manifestations are presented in Figure 2b. Interestingly, among the total cohort of patients followed, 30% did not experience a new-onset PRIS clinical manifestation after propofol therapy was started (Figure 3). However, for the 70% of patients who experienced one or more new-onset PRIS-associated clinical manifestation, 57.4% (410/710) experienced two or more manifestations. The cumulative average number of new-onset PRIS clinical manifestations, on a per-patient basis, when censored to the number of days that propofol was administered, increased each day over the first 10 days of propofol therapy (Figure 4).

## Discussion

Our study is the first large, prospective study to identify the incidence of PRIS in critically ill adults administered propofol for longer than 24 hours. Although other reports have tried to characterize PRIS and identify the incidence of this syndrome, the conclusions that can be drawn from these reports are limited due to their retrospective nature, the fact that they included either patients who were not critically ill or who received propofol for only a short duration [11,13,14,28,31,32,34,35,37,46,52,63]. Given the increasing number of often fatal case reports of PRIS that have been published involving critically ill adults, this study provides valuable insight into a complex and poorly characterized syndrome. Although our study suggests that the incidence of PRIS is low, the actual number of cases that occur in the United States each year may be substantial given that five million patients are admitted to an ICU each year and that propofol is the preferred sedative for critically ill adults by up to 80% of clinicians [64,65].

Our study also provides clinicians with valuable information towards the design of future controlled studies investigating PRIS. Based on the estimate of PRIS we identified in our study, and assuming a power of 80% and a *P *< 0.05, a future comparative (i.e., propofol vs. non-propofol) trial would require 2068 patients in each arm to detect a 70% relative decrease in the incidence of PRIS and 10,795 patients in each group to detect a 25% relative decrease in the incidence of PRIS.

PRIS was first defined by Bray in 1998 as a sudden onset of marked bradycardia, resistant to treatment, with progression to asystole plus one of the following: hyperlipidemia, fatty infiltration of the liver, severe metabolic acidosis, or muscle involvement with evidence of rhabdomyolysis or myoglobinuria [10]. Other definitions have been proposed that incorporate a combination of PRIS symptoms or just a single PRIS manifestation but a lack of consensus surrounding a definition for PRIS exists [17,60,63]. The definition chosen to define PRIS in our study is consistent with published PRIS case reports [10,57,59,60,66]. Among the 83 published PRIS case reports, the most common first-reported signs of PRIS are new-onset metabolic acidosis (86%) and cardiac dysfunction (88%) [2-57]. The occurrence of other manifestations is less frequent and includes new-onset rhabdomyolysis (45%), renal failure (37%), and hypertriglyceridemia (15%) [2-57]. Therefore, we feel that our definition of PRIS is both evidence-based and conservative. However, it must be acknowledged that there may be a wide variation in the true incidence of PRIS depending on how PRIS is defined in terms of either the number and/or type of PRIS-related clinical manifestations experienced by the patient.

Many differences exist between our cohort and published case reports [2-57]. For example, among the 71 published PRIS cases where the dose of propofol was provided, 86% received a dose exceeding 83 μg/kg/min whereas among the 11 patients in our cohort who developed PRIS, a dose this high was administered in only 18% [2,4,8,20,23,26,28,29,34,42-45,47,51,52,55-57,60,63,66,67]. This is surprising given the relatively high severity of illness of our cohort and the fact that 25% of the patients were admitted to a neurosurgical service - a population of patients that frequently requires propofol at high doses for a prolonged period of time [68]. It may be possible that the patients who developed PRIS were being administered far greater doses of propofol than was documented in the patient record given recent reports of intravenous smart pump practices demonstrating that propofol is the most likely drug to be delivered as a bolus in the ICU [65]. Another possible explanation for the low use (10%) of high-dose propofol in our overall cohort may relate to the fact that sedation guidelines advocating a maximum dose of propofol ranging from 60 to 83 μg/kg/min were in place at 10 of the 11 institutions who participated in the study and that a critical care pharmacist promoted these guidelines on a daily basis. The fact that the time from the start of propofol to the time PRIS was identified is longer in published PRIS case reports (median 2.5 days) than our study is likely attributable to the fact that monitoring for PRIS occurred immediately after propofol was started in our study and that the clinicians caring for our patients in our study may have been more aware of PRIS given the numerous recent publications surrounding it.

A third important difference between our 11 patients with PRIS and published case reports of adults developing PRIS relate to the fact that none of our PRIS patients developed rhabdomyolysis. Possible reasons for this disparity include the fact that the definition we chose for rhabdomyolysis in our study was frequently more stringent than the definition employed in the 45% of the adult case reports where rhabdomyolysis occurred. Other potential reasons for the lack of identified rhabdomyolysis in our 11 patients include the fact that CPK monitoring was not mandated as a part of our study and the fact that neurosurgical patients (an adult population with the highest incidence of rhabdomyolysis) only made up one-quarter of our cohort.

The mortality rate among our PRIS patients (18%) was markedly lower than that reported in published case reports or that predicted by their ICU admission APACHE II score (53%) [60,62,66,69]. Potential reasons for this discrepancy include the fact that only case reports resulting in the worse outcome (e.g. death) are usually published, that pediatric patients were excluded from our analysis (a population with worse outcomes from PRIS than adults), that only one-quarter of our cohort was admitted to a neurosurgical service (a population of patients who have a baseline mortality rate of more than 50% and who frequently require propofol at high doses for a prolonged duration), and that APACHE II score may overestimate patient mortality given the improvements in ICU care that have occurred over the 25 years since it was first validated [68,70,71].

Recent evidence suggests that PRIS may occur from an overlap of priming (i.e. baseline critical illness) and triggering (i.e. use of high-dose propofol) factors [60]. For example, a patient with cardiac dysfunction prior to the start of propofol therapy may be at greater risk for experiencing hypotension, renal failure and metabolic acidosis after propofol therapy is initiated. When we included patients who experienced PRIS manifestations both in the 24 hours prior to the start of propofol therapy and after propofol therapy was initiated, the incidence of PRIS increased to 4.7%. Although the incidence of PRIS is very unlikely to be as high as 4.7%, further research is required to determine the influence that a PRIS clinical manifestation present prior to the start of propofol therapy plays in causing PRIS.

There are a number of potential limitations to our study. By not evaluating a control group of patients receiving a non-propofol sedation regimen(s), it remains unclear if the clinical symptoms of PRIS that were identified were truly a result of propofol therapy or related to some other manifestation of critical illness and thus our reported incidence of PRIS may be greater than what truly exists. The specific cause for each PRIS-associated clinical manifestation (e.g. unexplained metabolic acidosis) was not investigated (e.g. additional diagnostic testing) outside of that which would occur in routine clinical practice. The incidence of PRIS may have been higher than our reported value if laboratory monitoring was required to determine PRIS manifestations such as rhabdomyolysis and hypertriglyceridemia. Finally, we did not mandate the discontinuation of propofol as a part of the study when PRIS was detected and thus cannot reliably estimate the resolution of PRIS in these situations.

## Conclusions

In summary, the incidence of PRIS in a heterogeneous population of critically ill adults prescribed propofol for more than 24 hours is approximately 1% and can occur soon after the initiation of propofol therapy and at low doses. In contrast to most of the published PRIS case reports, most of the patients in our cohort who developed PRIS survived and rhabdomyolysis did not occur. Data from both our study and previously published reports of PRIS suggest that PRIS may occur when propofol is administered at a low dose (<83 μg/kg/min) or for a short duration [11,13,14,28,31,32,34,35,37,39,46,52,63]. This suggests that clinicians should monitor patients for signs of PRIS from the time that propofol is initiated regardless of the propofol dose that is administered and over the entire course of propofol therapy. Based on the findings of our study, future controlled studies investigating PRIS will need to be large (at least 2,000 patients per arm). In addition, future studies will need to explore the mechanisms and risk factors associated with PRIS and investigate whether propofol manifests the derangements of critical illness more than other sedatives (e.g. benzodiazepines, dexmedetomidine).

## Key messages

• This study was the first to prospectively evaluate a large population of critically ill adults receiving longer-term propofol and to use an evidence-based and conservative definition for PRIS, and identified PRIS in 1.1% of patients.

• Compared with the 43 published case reports of PRIS in adults, our patients who developed PRIS developed it both faster after the start of propofol and at a lower propofol dose, had a lower mortality rate, and were less likely to experience rhabdomyolysis.

• Future comparative (i.e. propofol vs. non-propofol) trials surrounding PRIS will need to be large (from 2068 to 10,795 patients in each arm) depending on what the difference in PRIS between groups is deemed to be clinically significant.

## Abbreviations

APACHE: acute physiology and chronic health evaluation; CPK: creatinine phosphokinase; FDA: Food and Drug Administration; ICU: intensive care unit; PRIS: propofol-related infusion syndrome.

## Competing interests

The authors declare that they have no competing interests.

## Authors' contributions

JWD was responsible for the concept, acquisition and interpretation of data, manuscript preparation, and final manuscript approval. RJR and JFB were responsible for the acquisition and interpretation of data, manuscript preparation, and final manuscript approval. GS and RR were responsible for the interpretation of data, manuscript preparation, and final manuscript approval. JJF, PJK, SP, DY, EK, RX, ATG, PS, KA, PA, SV, and PG were responsible for the acquisition of data and final manuscript approval.
